# Development of dual-beamline photoelectron momentum microscopy for valence orbital analysis

**DOI:** 10.1107/S1600577524002406

**Published:** 2024-04-15

**Authors:** Kenta Hagiwara, Eiken Nakamura, Seiji Makita, Shigemasa Suga, Shin-ichiro Tanaka, Satoshi Kera, Fumihiko Matsui

**Affiliations:** a UVSOR Synchrotron Facility, Institute for Molecular Science, NishigoNaka 38, Myodaiji, Okazaki 444-8585, Japan; b SANKEN, Osaka University, Mihogaoka 8-1, Ibaraki, Osaka 567-0047, Japan; c The Graduate University for Advanced Studies (SOKENDAI), NishigoNaka 38, Myodaiji, Okazaki 444-8585, Japan; ESRF – The European Synchrotron, France

**Keywords:** photoemission spectroscopy, photoelectron momentum microscopy, electronic structure, atomic orbital, photon polarization

## Abstract

The soft X-ray photoelectron momentum microscopy (PMM) experimental station at the UVSOR Synchrotron Facility has been recently upgraded by additionally guiding vacuum ultraviolet (VUV) light in a normal-incidence configuration. PMM offers a very powerful tool for comprehensive electronic structure analyses in real and momentum spaces. In this work, a VUV beam with variable polarization in the normal-incidence geometry was obtained at the same sample position as the soft X-ray beam from BL6U by branching the VUV beamline BL7U. The valence electronic structure of the Au(111) surface was measured using horizontal and vertical linearly polarized (s-polarized) light excitations from BL7U in addition to horizontal linearly polarized (p-polarized) light excitations from BL6U. Such highly symmetric photoemission geometry with normal incidence offers direct access to atomic orbital information via photon polarization-dependent transition-matrix-element analysis.

## Abstract 2

The only photoelectron momentum microscopy experimental station at the UVSOR Synchrotron Facility has been developed by simultaneously using soft X-ray and vacuum ultraviolet beamlines. Atomic orbital analysis of the valence band structure of Au(111) was performed using normal-incidence photoemission geometry.

## 1. Introduction

Photoelectron momentum microscopy (PMM) is a new imaging-type photoelectron spectroscopy system that combines a photoemission electron microscopy (PEEM)-type objective lens and an energy-filtering hemispherical deflection analyzer (HDA) (Krömker *et al.*, 2008 ▸; Tusche *et al.*, 2015 ▸; Schönhense *et al.*, 2020 ▸). PMM maps the spatial distribution of photoelectrons in real space *I*(*E*_binding_, *x*, *y*), and surface topography and chemical composition can be imaged. By switching the operation mode of the objective lens, the angular distribution of photoelectrons can be captured in momentum space coordinates in the form of a 2D pattern *I*(*E*_binding_, *k*_*x*_, *k*_*y*_). The detailed valence band structure of the selected area can be analyzed. The detection efficiency is a few orders of magnitude higher than that of conventional angle-resolved photoelectron spectroscopy (ARPES) owing to an energy-filtering HDA in the case of the acquisition of 2D momentum distribution maps *I*(*E*_binding_, *k*_*x*_, *k*_*y*_). PMM has revolutionized the investigation of detailed electronic structures of materials and devices ranging from millimetres to tens of nanometres. By using both real- and momentum-space operation modes, PMM facilitates highly efficient space-resolved band structure measurement or ‘micro-spectroscopy’. So far, we have successfully visualized micrometre-scale local domain selective band structures (Matsui *et al.*, 2022 ▸; Hashimoto *et al.*, 2022 ▸).

Synchrotron radiation ranging from ultraviolet (UV), vacuum ultraviolet (VUV) light to soft and hard X-rays has been used for various purposes in photoelectron spectroscopy (Suga & Tusche, 2015 ▸; Suga *et al.*, 2021 ▸). Examples include (i) probing depth-dependent (from surface to bulk) valence band structure, (ii) *k*_*z*_ dispersion and 3D Fermi surface mapping by scanning photon energy, (iii) core-level X-ray photoelectron spectroscopy and diffraction, and (iv) resonant photoelectron spectroscopy near an element-selective specific core absorption edge. Some of these techniques can also be applied to spatial imaging using a PEEM. Scanning photon energy over certain absorption edges is called XAS-PEEM, and scanning kinetic energy over various electron core levels is called XPEEM. These approaches facilitate the spatial mapping of elements and are regarded as ‘spectro-microscopy’ (Locatelli & Menteş, 2015 ▸; Makita *et al.*, 2021 ▸).

PMM has been installed at the undulator-based soft X-ray beamline BL6U (Matsui *et al.*, 2020*a* ▸, 2023 ▸) of the UVSOR Synchrotron Facility (Ota *et al.*, 2022 ▸). To expand the available photon energies to the lower side, we have recently upgraded the PMM experimental station. It became feasible to branch and guide VUV light from BL7U to the same position on the sample excited by soft X-rays from BL6U. This beam from the BL7U branch with variable polarization can be introduced from the back of the first HDA; it passes through the PEEM objective lens and is normally incident on the sample. Currently, several PMMs are in operation at synchrotron facilities, such as the NanoESCA beamline at Elettra Sincrotrone Trieste (Wiemann *et al.*, 2011 ▸), DESY (Schönhense & Elmers, 2022 ▸) and BL27A2 at Taiwan Photon Source (Chuang *et al.*, 2024 ▸). The PMM at the UVSOR Synchrotron Facility is the first example of the implementation of a dual-beamline combination.

One advantage of PMM is that it captures wide momentum (*k*_∥_) space up to several Å^−1^, corresponding to all the photoelectrons emitted in the solid angle range up to θ = ±90° when the photon energy is less than 20 eV, which is sufficient to cover the full first Brillouin zone (BZ) of Au(111) ( 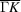 = 1.45 Å^−1^). In contrast, the acceptance angle of the typical ARPES analyzer is limited to θ = ±30°, corresponding to a maximum *k*_∥_ of approximately ±1 Å^−1^ for a photon energy of 20 eV.

PMM offers a fixed photoemission geometry, where the angle of photon incidence remains constant during the measurement without need to rotate the sample. In particular, rotating the sample and subsequently changing the angle of photon incidence in the case of typical ARPES during experiments inevitably varies the photoemission geometry and corresponding transition-matrix-element effect. Asymmetric photoemission geometry with grazing incidence induces asymmetric intensity distribution. These effects often hinder the detailed and reliable theoretical analysis of the obtained experimental results. Thus, a highly symmetric photoemission geometry with normal incidence in the PMM enables direct access to orbital information through photon-polarization-dependent transition-matrix-element analysis. Momentum-resolved photoelectron spectroscopy in the normal-incidence geometry was available in the previous DIANA analyzer (Daimon & Matsui, 2006 ▸; Matsui *et al.*, 2002 ▸, 2005 ▸), but a microscopic imaging capability was lacking. Currently, this measurement concept is only available with UVSOR PMM.

## 2. Beamline description

**Figure 1:**
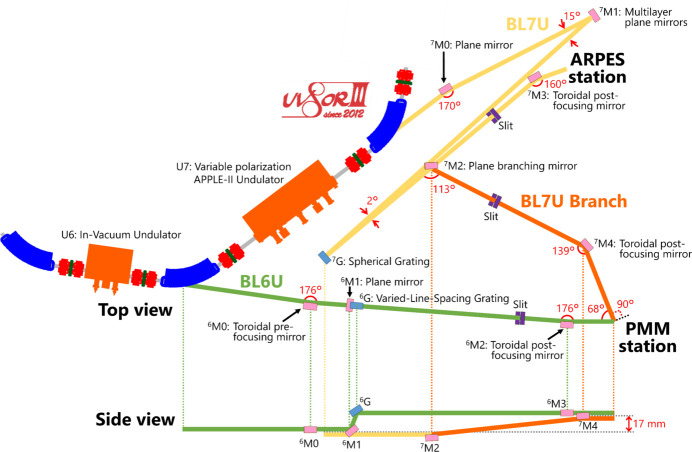
Schematic layout of beamlines BL6U, BL7U and the newly constructed BL7U branch at the UVSOR Synchrotron Facility. Top and side views are shown.

Fig. 1 ▸ illustrates the layout of the newly constructed BL7U branch with BL6U and BL7U. The in-vacuum undulator U6 installed as the light source for BL6U provides horizontal linearly polarized light. A variable-included-angle Monk–Gillieson mounting monochromator with varied-line-spacing plane grating ^6^G provides VUV to soft X-ray photons in the energy range *h*ν = 40–800 eV (Yamane *et al.*, 2019 ▸; Matsui *et al.*, 2020*a* ▸). The side view shows that the beam path height increases by 17 mm after reflection at the ^6^M1 mirror and the ^6^G grating. Accordingly, the beam focal point at the sample is higher than the height of the undulator center.

The variable-polarization APPLE-II-type undulator U7 of BL7U can produce horizontal/vertical linearly and right/left circularly polarized light. The undulator light is guided to the ^7^G spherical grating by the ^7^M0 and ^7^M1 plane mirrors and monochromated by the ^7^G grating. To obtain UV-to-VUV photons, a VUV near-normal-incident monochromator, namely a modified Wadsworth-type monochromator, was adopted. The appropriate selection of a multilayer plane mirror (^7^M1) out of eight mirrors and a grating (^7^G) out of three gratings provides optimized photons with the desired energy and polarization in the energy range *h*ν = 6–40 eV. A high-energy-resolution ARPES experimental station (SAMRAI beamline) (Kimura *et al.*, 2010 ▸) is available at the end of BL7U. Further details are given by Kimura *et al.* (2010 ▸).

The BL7U branch was designed to supply sufficient photon flux with both horizontally and vertically polarized light and match the focal points of BL6U and the BL7U branch at the same position on the sample. The retractable ^7^M2 plane mirror was installed after the ^7^G grating in this design. The ^7^M2 mirror guides the light to the branch, *i.e.* the PMM endstation. Aluminium was used for the ^7^M2 and ^7^M4 mirrors owing to its high reflectance in the low-energy region below *h*ν = 20 eV. The reflectance for the horizontally polarized light is lower than that for vertically polarized light and even becomes 0 in the case of the Brewster angle. To obtain sufficiently high-flux photons with horizontally polarized light at the BL7U branch and hold a workspace for BL7U users, a beamline arrangement was adopted with a reflection angle of 113° at the ^7^M2 mirror and 139° at the ^7^M4 mirror. As shown in the side view, after reflection on the ^7^M2 mirror the beam is reflected upward and becomes 17 mm higher at the ^7^M4 mirror than the undulator center. After reflection on the ^7^M4 mirror, the beam goes horizontally and the beam focal point of the BL7U branch matches that of BL6U. By inserting/retracting the ^7^M2 mirror, we can switch between the PMM experiment at the end of the BL7U branch and the ARPES experiment (Kimura *et al.*, 2010 ▸) at the end of BL7U.

**Figure 2:**
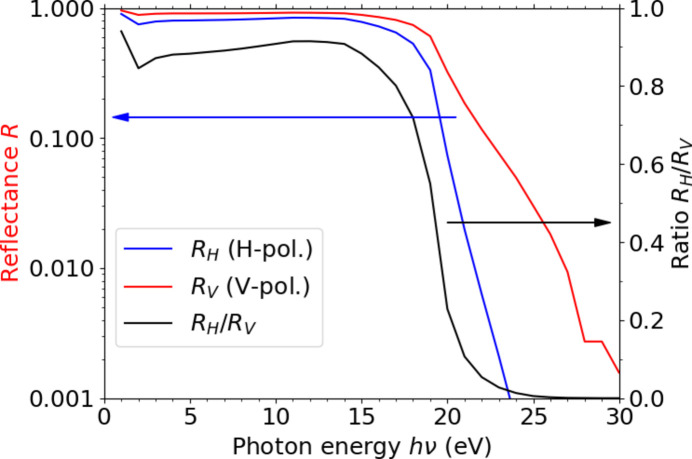
Simulation of the total reflectance for using the ^7^M2 and ^7^M4 aluminium mirrors for the adopted beamline arrangement as a function of photon energy. Reflectance values were obtained from experimental data of Rakić (1995 ▸) using the refractive index database (https://refractiveindex.info/). The reflectance for both horizontally polarized light (H-pol.) *R*_H_ and vertically polarized light (V-pol.) *R*_V_ is plotted against the left vertical axis. The ratio *R*_H_/*R*_V_ is plotted against the right vertical axis. *R*_H_ and *R*_V_ are nearly unity below *h*ν = 15 eV, and start to significantly decrease above *h*ν = 18 eV.

Fig. 2 ▸ shows a simulation of the total reflectance of the ^7^M2 and ^7^M4 aluminium mirrors for the adopted beamline arrangement as a function of photon energy. The total reflectance is calculated by multiplying the reflectance of the ^7^M2 and ^7^M4 mirrors. The reduction in intensity as a function of photon energy and polarization at the PMM station compared with the existing BL7U endstation can be estimated from the simulation data. The reflectance values were obtained from experimental data given by Rakić (1995 ▸) using the refractive index database (https://refractiveindex.info/). The reflectance for vertically and horizontally polarized light is approximately unity below *h*ν = 15 eV. This suggests that circularly polarized light can be obtained in addition to linearly polarized light. We are currently preparing a gap table for circular polarization for the APPLE-II-type undulator U7. The reflectance starts to decrease around *h*ν = 18 eV toward higher photon energies. The reflectance for horizontally polarized light decreases much more significantly than that for vertically polarized light. Although helicity is not ideal, we can still expect to obtain ellipsoidally polarized light for circular dichroism measurement in the photon energy range up to around 20 eV.

**Figure 3:**
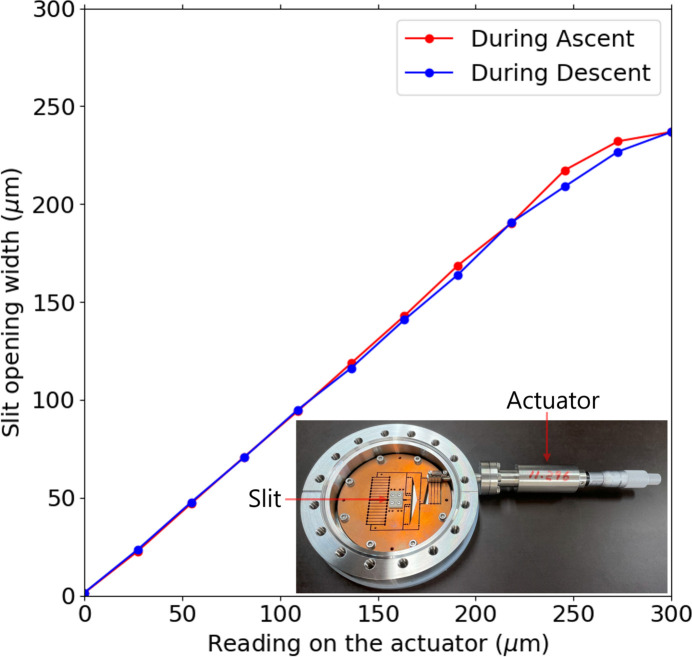
Flexure stage slit. The actual slit-opening width is plotted as a function of the reading on the actuator. The inset shows its photograph.

We installed an in-house-made flexure stage slit for the exit slit of the BL7U branch. As shown in Fig. 3 ▸, the actual slit-opening width ranged from 4 µm to 200 µm. The backlash of the slit was within several micrometres. The reproducibility of the slit-opening width was within the value of the backlash.

## 3. Setup of the photoelectron momentum microscope

**Figure 4:**
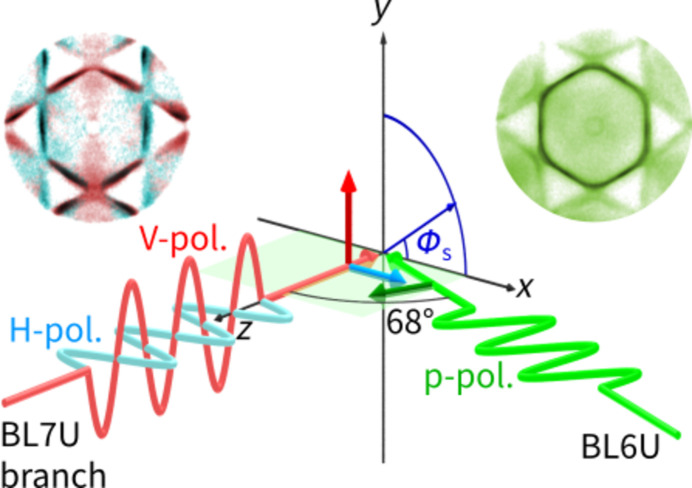
Experimental geometry of the PMM station with two beams. Linearly p-polarized light (p-pol.) from BL6U is incident in the *x*–*z* plane at an angle of 68° from the sample surface normal (*z*) axis. Here, the plane of incidence (*x*–*z* plane) is represented by the green horizontal plane. H-pol. and V-pol. enter from the back of the first HDA through the optical lens at the BL7U branch in the normal-incidence geometry. Any sample can be rotated in-plane at an angle ϕ_s_. The two insets show the measured photoelectron momentum patterns of the Au(111) surface with various photon polarizations.

Fig. 4 ▸ illustrates the experimental geometry of PMM. The sample surface is in the *x*–*y* plane. The photon beam from BL6U is incident in the *x*–*z* plane at an angle of 68° from the sample surface normal (*z*) axis and is horizontally polarized in the *x*–*z* plane. The light polarization, *i.e.* the electric vector, parallel to the plane of incidence is denoted as p-polarization, whereas the polarization perpendicular to the plane of incidence is denoted as s-polarization. Thus, we refer to the beam from BL6U as linearly p-polarized light (p-pol.).

The photon beam from BL7U passes along the surface normal axis. Linearly polarized light in the *x*–*z* or *y*–*z* plane is available at the PMM experimental station. In such a normal-incidence geometry, the polarization vector is always perpendicular to the surface normal. Hereafter, we refer to the light from the BL7U branch polarized in the *x*–*z* plane as horizontally polarized light (H-pol.), and the light polarized in the *y*–*z* plane as vertically polarized light (V-pol.). Recently, a PMM with a single HDA (Matsui *et al.*, 2020*a* ▸, 2023 ▸) has been upgraded to that with double HDAs. For normal-incidence experiments, the entrance of the first HDA is set to be open so that the beam can pass through the optical lens of the PMM and arrive at the sample position. A smaller slit is placed at the exit of the first HDA instead (Tusche & Kirschner, 2006 ▸).

**Figure 5:**
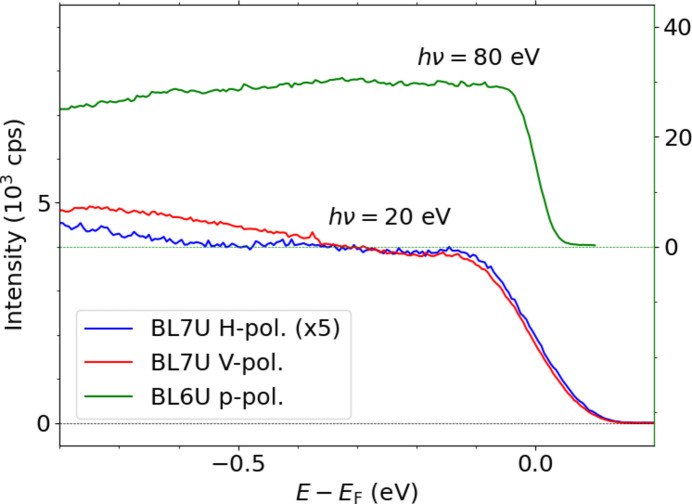
Photoelectron spectra of Au(111). The spectra were measured using H-pol. and V-pol. at *h*ν = 20 eV from the BL7U branch. The spectrum was also measured using p-pol. at *h*ν = 80 eV from BL6U. The spectra obtained by the BL7U (BL6U) light are plotted against the left (right) vertical axis in counts per second (cps).

Fig. 5 ▸ shows the photoelectron spectra of the Au(111) surface measured using horizontally and vertically polarized light from the BL7U branch and p-polarized light from BL6U. The Au(111) surface was cleaned by repeating the cycle of Ar^+^ sputtering and annealing at 550°C. The detailed settings used in this work are as follows. The sample temperature was below 10 K. The pass energy (*E*_pass_) was set to 50 eV for all measurements. The photon energy from the BL7U branch was set to 20 eV. The sizes of the entrance and exit slits of the first HDA were 6 mm-diameter and 1 mm-diameter, respectively, and those of the second HDA were 6 mm-diameter and 1 mm  ×  3 mm, respectively. The photon energy from BL6U was set to 80 eV. The sizes of the entrance and exit slits of the first HDA were 0.5 mm  ×  1 mm and 4 mm-diameter, respectively, and those of the entrance and exit slits of the second HDA were 4 mm-diameter and 0.5 mm × 3 mm, respectively. The spectra near the Fermi level were fitted by the Fermi–Dirac function. The energy resolution was estimated to be approximately 90 and 45 meV for the measurement of BL7U and BL6U, respectively. Because spectral broadening due to thermal vibrations and photon energy width resolution on the order of several meV are negligible in this case, the energy resolution was mainly determined using the pass energy and slit size. Notably, our PMM system currently achieved a momentum resolution of 0.012 Å^−1^ and an energy resolution of 22.74 meV at a pass energy of *E*_pass_ = 20 eV with the highest resolution setting (Matsui *et al.*, 2020*a* ▸). A typical established PMM realizes a momentum resolution of 0.005 Å^−1^ and an energy resolution of 12 meV at *E*_pass_ = 15 eV (Tusche *et al.*, 2015 ▸), 0.005 Å^−1^ and 4.2 meV at *E*_pass_ = 8 eV (Schönhense *et al.*, 2020 ▸, 2021 ▸).

According to the simulation of the reflectance of the aluminium mirrors shown in Fig. 2 ▸, the ratio of the reflectance of horizontally and vertically polarized light *R*_H_/*R*_V_ is 0.229 at *h*ν = 20 eV. The photoelectron spectrum measured with horizontally polarized light was about one-fifth of that measured with vertically polarized light as recognized in Fig. 5 ▸. A ratio of their photoelectron intensity approximately follows the designed value. These results confirm the successful guidance of the BL7U branched linearly polarized light onto the sample in the PMM experimental station.

## 4. Photon-polarization-dependent photoelectron momentum distribution of Au(111)

**Figure 6:**
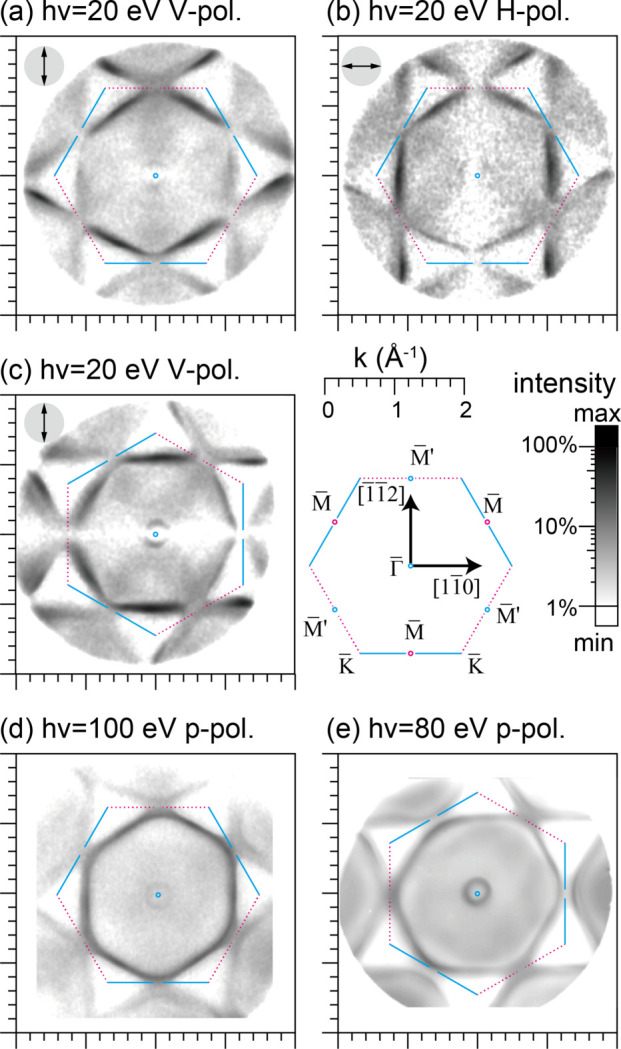
2D momentum (*k*_*x*_, *k*_*y*_) distribution of the photoelectron intensity of the Au(111) surface at the Fermi level plotted on a logarithmic contrast scale. Measurements were performed with V-pol. at *h*ν = 20 eV (*a*, *c*), H-pol. at *h*ν = 20 eV from the BL7U branch (*b*) and p-pol. at *h*ν = 100 eV from BL6U (*d*). (*e*) High-resolution measurement taken using p-pol. at *h*ν = 80 eV from BL6U. In (*c*) and (*e*), the sample is in-plane rotated by ϕ_s_ = −30°. The upper left insets describe the experimental geometry of the electric vector of the incident light.

Fig. 6 ▸ shows the 2D momentum (*k*_*x*_, *k*_*y*_) distribution of the photoelectron intensity of the Au(111) surface at the Fermi level with an energy window of ±50 meV taken at *h*ν = 20 eV (*a*, *b*, *c*) and 100 eV (*d*). Measurements were performed using vertically polarized light (V-pol.) at *h*ν = 20 eV (*a*, *c*), horizontally polarized light (H-pol.) at *h*ν = 20 eV from the BL7U branch (*b*), and p-polarized light at *h*ν = 100 eV from BL6U (*d*). Fig. 6 ▸(*e*) shows the measurement of the high-resolution settings obtained using p-polarized light at *h*ν = 80 eV from BL6U. The estimated momentum resolution for the measurement in Fig. 6 ▸(*e*) was 0.03 Å^−1^. The acquisition times in Figs. 6 ▸(*a*, *b*, *c*), Fig. 6 ▸(*d*) and Fig. 6 ▸(*e*) were 36, 18 and 1500 s, respectively. The surface BZ center 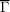 excited by *h*ν = 20 and 100 eV corresponds approximately to the high-symmetry Γ_111_ [= 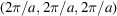 ] and Γ_222_ [= (4π/*a*, 4π/*a*, 4π/*a*)] points of the bulk BZ in (*k*_*x*_, *k*_*y*_, *k*_*z*_), respectively, by assuming an inner potential from the vacuum level of 10.6 eV (Au lattice constant *a* = 4.079 Å) (Matsui *et al.*, 2020*b* ▸, 2022 ▸). Notably, in Figs. 6 ▸(a)–6(*c*), we captured all photoelectrons emitted up to the emission angle θ = ±90° (or within the emission cone of 2π steradian) at *h*ν = 20 eV by the PMM system, covering the maximum wide *k*_∥_ space.

The surface structure of the Au(111) crystal is threefold symmetric. The mirror plane of the Au(111) crystal ( 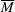 – 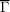 – 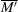 ) was aligned in the *y*–*z* plane, *i.e.* perpendicular to the plane of incidence (*x*–*z* plane) in the BL6U configuration. The electric vector of the incident light was aligned in the 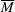 – 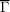 – 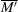 direction for Fig. 6 ▸(*a*) and the 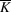 – 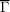 – 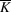 direction for Fig. 6 ▸(*b*).

In Fig. 6 ▸(*c*), we rotated the sample in-plane by ϕ_s_ = −30° (clockwise 30°) and measured it using vertical polarization. Here, the photon polarization vector was set along the 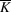 – 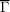 – 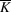 direction, *i.e.* the linear polarization geometry with respect to the sample was the same as for Fig. 6 ▸(*b*). In short, experiments with two different photoemission geometries can also be performed by rotating the sample [Fig. 6 ▸(*a*) versus Fig. 6(*c*)] instead of switching the photon polarization and fixing the sample orientation [Fig. 6 ▸(*a*) versus Fig. 6(*b*)]. Our PMM experimental station allows in-plane sample rotation from ϕ_s_ = −90° to 90°, offering detailed measurements with tunable linear polarization geometries.

The Shockley surface state is centered at the 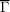 point as a small circular contour. The Shockley surface state mainly comprises 6*s* and 6*p*_*z*_ orbitals. In the normal-incidence geometry, the transition-matrix element from the initial *s* and *p*_*z*_ orbitals becomes 0 at the photoemission direction orthogonal to the excited electric vector and small at a small emission angle θ. These features indicate very weak intensity for normal incident light. To capture the Shockley surface state, we opened the slit of the HDAs, obtained a higher photoelectron transmission efficiency, and sacrificed the resolution. We successfully detected the Shockley surface state for the normal incident light as shown in Figs. 6 ▸(*a*)–6(*c*) plotted on a logarithmic contrast scale. As shown in Fig. 6 ▸(*a*) and most clearly recognized in Fig. 6 ▸(*c*), the intensity of the Shockley surface state is suppressed at the *k*_*y*_ = 0 line when using normal-incident vertically polarized light from the BL7U branch. As shown in Fig. 6 ▸(*b*), it is suppressed at the *k*_*x*_ = 0 line in the case of horizontal polarized light. Thus, our observation discussed here is consistent with the expected behavior, indicating that the Shockley surface state is mainly composed of 6*s* and 6*p*_*z*_ orbitals. In Fig. 6 ▸(*d*), the intensity suppression of the Shockley surface state is not observed when using p-polarized light from BL6U because of the grazing incidence.

One can see the features corresponding to the cross section of the Fermi sphere of the bulk gold crystal: the nearly hexagonal contour centered at the 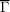 point and the so-called neck features located around the 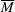 point, which connects the Fermi surfaces of the neighboring BZs. Fig. 6 ▸(*d*) shows the result obtained using p-polarized light at *h*ν = 100 eV from BL6U. Here, we can find the left–right asymmetry of the intensity with respect to the *k*_*x*_ = 0 line. This intensity asymmetry, shown in Fig. 6 ▸(*d*), is caused by the asymmetric photoemission geometry with grazing-incident p-polarized light and reflects the linear dichroism in the angular distribution (LDAD) (Cherepkov & Schönhense, 1993 ▸; Chernov *et al.*, 2015 ▸). The tilted electric vector of p-polarized light breaks the mirror symmetry with respect to the *y*–*z* plane, *i.e.* the orthogonal plane to the plane of incidence, enhancing intensity asymmetry. The term dichroism refers to the different response of a system to p-polarized light coming from the *x* and −*x* directions in the *x*–*z* plane at a definite angle. Importantly, the observed LDAD is caused solely by mirror symmetry breaking due to the incident light because the crystal mirror plane, *i.e.* the 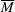 – 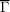 – 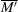 direction of the Au(111) surface BZ, is aligned perpendicular to the plane of incidence.

Figs. 6 ▸(*a*) and 6(*b*) show the results obtained using vertically and horizontally polarized light at *h*ν = 20 eV from the BL7U branch. Owing to the normal-incidence geometry, the measured intensity is symmetric with respect to the *k*_*x*_ = 0 line without the LDAD effect of the incident light in contrast to the result shown in Fig. 6 ▸(*d*), facilitating atomic orbital analysis. Normally, atomic orbital analysis is performed using polarization dependence as follows: orbitals with even (odd) symmetry can be detected by p- (s-) polarized light (Daimon *et al.*, 1999 ▸; Damascelli *et al.*, 2003 ▸). However, this criterion is applied only to photoelectron intensities in the corresponding mirror plane (Daimon *et al.*, 1999 ▸). Usually, ARPES only focuses on the band structure in such a symmetrical plane. Because we captured the band structure over the entire BZ by PMM, we can analyze the atomic orbitals of the Au(111) surface using the calculated 2D distribution of the transition-matrix element from the fundamental atomic orbitals (Daimon *et al.*, 1999 ▸). Here, we assumed that the density of states near the Fermi level is dominated by Au 6*p* orbitals (Matsui *et al.*, 2020*b* ▸). Starkly different intensity distributions of the bulk Fermi surface contour can be found between Figs. 6 ▸(*a*) and 6(*b*), as can be observed from the dichroism diagram shown in the upper left inset of Fig. 4 ▸. On the *k*_*x*_ = 0 line, the intensity is suppressed for horizontal polarization [Fig. 6 ▸(*b*)] and enhanced for vertical polarization [Fig. 6 ▸(*a*)], indicating that the states around 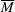 and 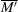 on the *k*_*x*_ = 0 line mainly comprise *p*_*y*_ or *p*_*z*_ orbitals. The lower and upper sides of the nearly hexagonal contour exhibit stronger intensity for vertical polarization [Fig. 6 ▸(*a*)] and finite intensity for horizontal polarization [Fig. 6 ▸(*b*)], implying that these states mainly comprise *p*_*x*_, *p*_*y*_ or *p*_*z*_ orbitals. The intensity of the nearly hexagonal contour along *k*_*x*_ = ±1 Å^−1^ parallel to the *k*_*y*_ axis is enhanced for horizontal polarization [Fig. 6 ▸(*b*)] and suppressed for vertical polarization [Fig. 6 ▸(*a*)]. This suggests that these states are mainly composed of *p*_*x*_ or *p*_*z*_ orbitals. By taking these results into account, the nearly hexagonal contour is determined to be composed of mainly in-plane *p* orbitals pointing outward from the 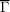 point and the *p*_*z*_ orbital. This result is similar to the atomic orbitals composing the bulk Fermi surface of the Cu(001) surface (Matsui *et al.*, 2005 ▸).

## 5. Conclusion

We successfully branched the VUV beamline BL7U and used both VUV and soft X-ray beams in the PMM experimental station. It is now possible to irradiate normal-incidence VUV and grazing-incidence soft X-rays at the same sample position, orientation and temperature. When measuring the Au(111) band structure using grazing-incident light from BL6U, we observed asymmetry in the photoelectron intensity. Such intensity asymmetry due to the asymmetric photoemission geometry can be eliminated using the highly symmetric photoemission geometry with normal-incident light from BL7U, facilitating the atomic orbital analysis of valence electronic states. The different intensity distributions between horizontal and vertical polarizations indicate that the Fermi surface counter of Au(111) mainly comprises in-plane *p* orbitals pointing outward from the 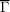 point. Furthermore, our PMM experimental station can adjust any linear polarization geometry with respect to the sample through in-plane sample rotation. Our PMM experimental station can be used not only for the atomic orbital analysis, as demonstrated in this work, but also for comprehensive and in-depth electronic structure analyses in real and momentum spaces owing to wide-energy photons. By combining momentum-selected PEEM known as the dark-field imaging technique (Matsui *et al.*, 2022 ▸; Hashimoto *et al.*, 2022 ▸) with photon polarization, exotic orbital structures in real space such as charge–orbital order can be visualized (Mizokawa *et al.*, 2016 ▸). Detailed analysis of the electronic structure of ordered molecular adsorbates is another important scientific goal (Puschnig *et al.*, 2009 ▸; Iwasawa *et al.*, 2022 ▸; Nozaki & Konishi, 2024 ▸). Real-space imaging by utilizing circularly polarized light in the normal-incidence geometry allows so-called magnetic circular dichroism (MCD)-PEEM, visualizing perpendicular ferromagnetic domains (Locatelli & Menteş, 2015 ▸; Taniuchi *et al.*, 2015 ▸). Circular dichroism in the angular distribution (CDAD) (or in the band structure) by utilizing the normal incidence eliminates the contribution of the experimental geometry-induced asymmetry to CDAD (Schönhense, 1990 ▸) which necessarily occurred in the grazing-incidence geometry. Thus, the CDAD in the normal incidence can be a powerful probe for, for example, chiral crystals (Fecher *et al.*, 2022 ▸).
